# Psychological Profile of Bariatric Surgery Candidates: A Cross‐Sectional Study

**DOI:** 10.1002/brb3.71750

**Published:** 2026-09-06

**Authors:** Şafak Yalçin Şahiner, Dilek Baysal, Hakkı Polat, Abdurrahim Bakirhan

**Affiliations:** ^1^ Department of Psychiatry Faculty of Medicine Ankara University Ankara Türkiye; ^2^ Combating for Addiction Application and Research Center Ankara University Ankara Türkiye; ^3^ METU Research Park Middle East Technical University Ankara Türkiye; ^4^ Department of Psychiatry Faculty of Medicine İnönü University Malatya Türkiye

**Keywords:** bariatric surgery, obesity, psychological profile, psychopathology

## Abstract

**Introduction:**

This study aimed to examine the relational roles of childhood trauma, parental care, and coping strategies in bariatric surgery candidates with respect to depressive symptoms and general psychopathology levels in adulthood, employing a comprehensive theoretical model.

**Methods:**

The study sample comprised 118 bariatric surgery candidates (mean age = 37.55 ± 10.36; 88.0% female) who had been referred to a clinical center and received a diagnosis of clinical obesity. Participants completed the Sociodemographic and Clinical Information Questionnaire, CTQ‐SF, COPE, SF‐36, RSE (Parental Interest Subscale), BDI, and SCL‐90‐R.

**Results:**

No statistically significant association was observed between body mass index and any of the psychosocial variables included in the study. The model evaluating predictors of general mental health symptoms accounted for 66% of the total variance. Statistically significant predictors within this model included depression, childhood trauma, coping strategies, and parental interest. In a separate model predicting depression, parental interest emerged as the sole statistically significant predictor. Notably, 83.8% of participants reported a history of at least one prior suicide attempt.

**Conclusion:**

These findings underscore the importance of systematically assessing these factors to inform and enhance personalized intervention strategies for bariatric surgery candidates.

## Graphical abstract

The present study examined the psychological profile of bariatric surgery candidates, revealing that early childhood trauma, parental neglect, and coping strategies were associated with adult psychopathology. No direct relationship was identified between BMI and psychological findings, underscoring the necessity of mandatory psychiatric screening before surgical intervention.

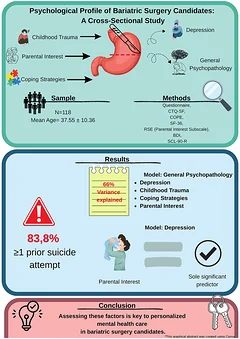

## 1 Introduction

Obesity has attracted considerable attention as a major public health concern due to its association with numerous comorbid conditions, high morbidity rates, and the substantial economic burden it imposes. According to the World Obesity Federation's 2024 Atlas, published in 2025, the global prevalence of clinical obesity has increased by 115% since 2010 and is projected to rise from 24 million to 1.13 billion individuals by 2030. Furthermore, obesity has been identified as a contributing factor to premature mortality among individuals with chronic diseases such as diabetes, hypertension, and cardiovascular disorders, accounting for approximately 1.3 million deaths worldwide (World Obesity Federation 2025). Notably, this figure exceeds the number of deaths caused by traffic accidents, underscoring the severity of the issue.

The causes of obesity are as complex as its consequences. Among these, the most prominent factors include childhood trauma, parental attitudes, and overall mental health problems. Empirical evidence has demonstrated a close association between obesity and the presence of childhood trauma, indicating that individuals with obesity more frequently report a history of adverse experiences. These experiences have been shown to mediate difficulties in coping strategies and to contribute to deterioration in both physical and psychological well‐being (Spirou et al. 2022; Offer et al. 2022; Danese and Tan 2014). When the close relationship between emotional eating and the risk of developing obesity is considered, it becomes evident that stressful life events and childhood adversities play a substantial role. Such negative life experiences may impair problem‐solving abilities and consequently lead to maladaptive behaviors, including eating disorders and obesity (Ben‐Porat et al. 2023).

Adverse life events and traumatic experiences in the past may lead to difficulties in emotion regulation during adulthood. This indicates that experiences in childhood do not remain confined to the past but can permeate behavioral patterns across the lifespan. Among clinically obese individuals who experience difficulties in emotion regulation, a greater vulnerability to depression is more frequently observed (Di Nicola et al. 2025). Consequently, depression can be considered not only a cause but also a consequence of maladaptive coping behaviors such as overeating and substance use (Orak et al. 2023).

In the long term, one of the most significant factors contributing to good mental health and strong coping strategies is the attitude and involvement of parents during childhood. Parenting styles characterized by neglectful behaviors in this period may intensify lifelong feelings of helplessness experienced both in childhood and adulthood, thereby leading to problems related to eating behaviors (Chung et al. 2015).

In a meta‐analysis examining impaired psychological well‐being as a proposed cause of obesity, a strong association was identified between obesity during childhood and adolescence and depression (Quek et al. 2017). Obesity negatively affects individuals' perceptions of their overall health, which may, in turn, lead to the development of psychopathology. A study has demonstrated that women with clinical obesity experience impairments in psychosocial functioning, suggesting that obesity can lead to both physical and psychological deterioration (Mond et al. 2007). As a result, obese individuals experience a decline in their quality of life. Moreover, not only the presence of obesity itself but also the prolonged and challenging nature of its treatment process may impose a psychological burden on individuals and intermittently disrupt their overall health perception (Giel et al. 2013).

The aim of this study is to examine, using a comprehensive and cross‐sectional model, the roles of early‐life and relational factors—such as childhood trauma, parental care, and coping strategies—in bariatric surgery candidates in relation to depressive symptoms and overall levels of psychopathology in adulthood. Furthermore, the study aims to empirically assess whether there is a relationship between these psychological and social variables and body mass index (BMI). Given the considerable diversity of psychological and relational constructs among bariatric surgery candidates and the relative scarcity of studies jointly modeling these variables, our study was designed as an exploratory investigation. Accordingly, correlational and regression‐based statistical analyses were employed to identify patterns and candidate associations among childhood trauma, parental care, coping strategies, depressive symptoms, psychological distress, and BMI. In this way, the study is thought to contribute to the literature by informing future targeted and hypothesis‐driven research.

## 1 Materials and Methods

Our study was conducted at the Endocrinology and Metabolic Diseases outpatient clinic of Ankara Numune Training and Research Hospital, involving individuals aged 18–65 who had previously been diagnosed with “Overweight and Obesity” according to ICD‐10 criteria by physicians and had been scheduled for obesity surgery. A cross‐sectional research design was adopted in our study. As this study followed an exploratory design, no directional hypotheses were tested. Correlation and regression analyses were applied to assess the relationships among the study variables.

Participants consisted of individuals who were referred to the Psychiatry Clinic of Ankara Numune Training and Research Hospital for preoperative consultation for obesity surgery in 2019. The data collection period lasted from April 2019 to December 2019. The study was conducted in accordance with the principles of the Helsinki Declaration and was approved by the Clinical Research Ethics Committee of Ankara Numune Training and Research Hospital (Approval number: E‐19‐2665, Date: April 18, 2019).

Initially, 120 patients with obesity referred for psychiatric consultation were enrolled; two declined consent, resulting in a final sample of 118 participants. After providing informed consent, participants were assessed face‐to‐face by the researcher in a private setting. The assessment scales were administered in a single session lasting approximately 30–40 min.

The inclusion criteria were an ICD‐10 diagnosis of overweight or obesity, age 18–65 years, literacy, Turkish proficiency, ability to complete the assessment scales, and provision of informed consent. Exclusion criteria included intellectual disability, dementia, psychotic or organic mental disorders, illiteracy, age outside the specified range, and refusal to participate. Eligibility was initially screened using hospital records and confirmed through medical file review. At the study site, patients recommended for bariatric surgery by the Endocrinology and Metabolic Diseases clinic were routinely referred for preoperative psychiatric evaluation.

*Sociodemographic and Clinical Information Questionnaire*: This questionnaire was developed by the researchers to collect data on sociodemographic and clinical characteristics. It was completed by the participants.

*Childhood Trauma Questionnaire–Short Form (CTQ‐SF)*: Originally developed by Bernstein et al. in 1994 as a 70‐item instrument, the scale was later revised into a 28‐item short form by the same authors (Bernstein et al. 1994, 2003). It is a self‐report measure based on a 5‐point Likert scale and consists of five subscales: *emotional abuse*, *physical abuse*, *sexual abuse*, *emotional neglect*, and *physical neglect*. The Turkish validity and reliability study of the scale was conducted by Şar et al. in 2012. The Cronbach's *α* coefficient indicating internal consistency was reported as 0.93, and the Guttman split‐half coefficient was 0.97 (Şar et al. 2012).

*Coping Orientation to Problems Experienced Inventory (COPE)*: The COPE inventory was originally developed by Carver et al. in 1989 and later revised to a 40‐item self‐report scale by Zuckerman and Gagné in 2003 (Carver et al. 1989; Zuckerman and Gagne 2003). The Turkish adaptation and reliability study was conducted by Dicle and Ersan in 2015, yielding a Cronbach's *α* coefficient of 0.766 (Dicle and Ersanlı 2015).

*The 36‐Item Short Form Survey (SF‐36)*: The scale was developed to assess individuals' health‐related quality of life and perceptions. It was created by Ware and Sherbourne in 1992, consists of 36 items, and is a self‐report measure (Ware and Sherbourne 1992). The Turkish validity and reliability study was conducted by Koçyiğit et al. in 1999, with Cronbach's *α* coefficients for the subscales ranging from 0.73 to 0.76 (Koçyiğit et al. 1999).

*Rosenberg Self‐Esteem Scale (RSE)—Parental Interest Subscale*: Developed by Rosenberg in 1965 to assess self‐esteem, this is a self‐report scale consisting of 12 subdomains. One of these, the “Parental Interest” subscale (Items 49–55), includes seven items (Rosenberg 1965). In the present study, only this subscale was used to evaluate parental interest. The Turkish adaptation and validity‐reliability study of the scale was conducted by Çuhadaroğlu in 1986, with a Cronbach's *α* coefficient of 0.76 (Çuhadaroğlu 1986).

*Beck Depression Inventory (BDI)*: Developed by Beck et al. in 1961 to assess the risk of depression and the severity of depressive symptoms, this self‐report scale uses a 4‐point Likert format and consists of 21 items (Beck et al. 1961). The Turkish validity and reliability study was carried out by Hisli in 1988, and the Cronbach's *α* coefficient was reported as 0.80 (Hisli 1988).

*Symptom Checklist‐90‐Revised (SCL‐90‐R)*: Developed by Derogatis in 1977 as a revision of the Hopkins Symptom Checklist, this 5‐point Likert‐type self‐report scale is used for psychiatric screening and consists of 90 items in total (Derogatis et al. 1976). The Turkish validity and reliability study was performed by Dağ in 1991, with a Cronbach's *α* coefficient of 0.97 (Dağ 1991).

### 1.1 Statistical Analysis

All statistical analyses were conducted using R (Version 4.2.2). Before the analyses, the dataset was screened for missing values and outliers. Descriptive statistics (mean, standard deviation, minimum, and maximum) were calculated for all study variables. Skewness and kurtosis values, as well as the Shapiro‒Wilk test, were examined to evaluate normality. Although some variables showed deviations from normality according to the Shapiro‒Wilk test, skewness and kurtosis values were within acceptable limits, and parametric tests were considered appropriate.

Pearson correlation analysis was conducted to examine the relationships between variables. Multiple linear regression analyses were performed to identify the predictors of psychological symptoms (SCL‐90‐R) and depression. In Model 1, psychological symptoms (SCL‐90‐R) were entered as the dependent variable, while depression, COPE Total, Childhood Trauma Total, SF‐36 General Health, and Parental Interest were entered as independent variables. In Model 2, depression was entered as the dependent variable, while COPE Total, Childhood Trauma Total, SF‐36 General Health, and Parental Interest were entered as independent variables. Model fit was evaluated using *R* ^2^, adjusted *R* ^2^, *F* statistics, and corresponding *p* values. Multicollinearity was assessed using variance inflation factor (VIF) values. A significance level of *p* < 0.05 was used for all analyses. In addition, missing values were handled using listwise deletion (complete‐case analysis), which is the default behavior of the *lm()* function in base R. Consequently, the final sample size differed across regression models (Model 1: *n* = 75; Model 2: *n* = 87).

## 2 Results

### 2.1 Clinical and Demographic Characteristics of the Sample

The study comprised 118 patients diagnosed with clinical obesity. The mean age of the participants was 37.55 years (SD = 10.36, range = 18–58). The mean BMI was 45.47 kg/m^2^ (SD = 6.33), and the mean duration of education was 9.39 years (SD = 4.16). The mean duration of obesity was 18.57 years (SD = 10.22). The mean duration of cigarette use was 51.79 months (SD = 106.39), and the mean duration of substance use was 1.03 months (SD = 11.09).

Of the participants, 88.0% were female and 12.0% were male. The majority of respondents were married (59.0%) and unemployed (64.1%). With regard to their occupation, 63.2% of the subjects were not in gainful employment, 24.6% were self‐employed, and 9.6% were employed by the state. The prevalence of substance use was reported by 11.1% of the participants, and 35.9% of the participants were current smokers. The majority of respondents lived with their families (95.7%) and resided in urban areas (97.4%). With respect to socioeconomic status, 45.3% of the sample reported a low income, 24.8% reported a very low income, and 6.0% reported a high income.

A family history of obesity was documented in 97.4% of the sample, predominantly among first‐degree relatives (65.0%). A history of suicide attempt was reported by 83.8% of participants, while parental loss was noted in 27.4%. A comorbid medical condition, most frequently ankylosing spondylitis, was present in 12.8% of cases. The majority (76.9%) of the subjects were not using regular medication.

With respect to obesity‐related and lifestyle factors, 52.1% of subjects reported a triggering factor for obesity, and 60.7% noted reduced physical activity. Almost half of the subjects (44.8%) reported previous surgery or illness related to obesity, while 35.0% associated obesity onset with pregnancy. Among the female subjects, 16.2% were classified as postmenopausal. Approximately 27.4% had a history of smoking cessation, and 24.8% reported weight changes related to marriage or divorce.

The survey revealed that 38.8% of the subjects had previously attempted dieting, 24.1% had undergone cavitation procedures, and 10.3% had undergone a combination of diet and exercise interventions. The survey revealed that 52.1% of the participants were currently engaged in a diet, while 47.9% were either participating in or had previously discontinued physical exercise. A total of 38.5% of the participants had requested a psychiatric consultation for obesity, and 26.5% of these individuals reported the use of anti‐obesity medication.

Table 1 presents the descriptive statistics of the study variables. Table 1 presents descriptive statistics and normality results for the study variables. The mean BMI of the participants was 45.47 (SD = 6.33). The mean scores were 150.17 (SD = 21.55) for coping, 49.16 (SD = 14.27) for childhood trauma, 93.89 (SD = 65.88) for psychological symptoms (SCL‐90‐R), 16.36 (SD = 10.93) for depression, 92.96 (SD = 9.19) for general health (SF‐36), and 16.52 (SD = 7.72) for parental interest.

**TABLE 1:** Descriptive statistics for study variables (*n* = 118).

Variable	*N*	Min	Max	*M*	SD	Skewness	Kurtosis	Shapiro‒Wilk *p*
BMI	114	25.95	60.61	45.47	6.33	−0.11	3.72	0.009
COPE Total	116	69.00	216.00	150.17	21.55	−0.63	5.32	< 0.001^**^
Childhood Trauma	118	28.00	109.00	49.16	14.27	1.15	5.04	< 0.001^**^
SCL‐90‐R	103	0.00	282.00	93.89	65.88	0.65	2.52	< 0.001^**^
Depression	113	0.00	45.00	16.36	10.93	0.64	2.55	< 0.001^**^
SF‐36 General Health	113	68.00	130.00	92.96	9.19	0.99	6.04	< 0.001^**^
ACY	92	0.00	60.00	16.52	7.72	1.91	12.38	< 0.001^**^

** *p* < 0.01 statistically significant.

**Skewness** and kurtosis values indicated that most variables were within acceptable limits for normality (±1.5), although parental interest showed higher skewness and kurtosis values. The Shapiro–Wilk test indicated significant deviations from normality for several variables. However, parametric analyses were used because skewness and kurtosis values were generally within acceptable ranges and regression analysis requires normality of residuals rather than variables.

The internal consistency of the scales used in this study was examined using Cronbach's *α* coefficients. The results showed that Cronbach's *α* values ranged between 0.78 and 0.98, indicating that all scales had acceptable to very high reliability. Specifically, the SCL‐90‐R (*α* = 0.98), the Childhood Trauma Scale (*α* = 0.96), and the COPE Scale (*α* = 0.94) demonstrated very high internal consistency. The BDI (*α* = 0.90), SF‐36 (*α* = 0.88), and the Rosenberg Self‐Esteem Scale (*α* = 0.82) showed good reliability. The ACY Scale had a Cronbach's *α* value of 0.78, indicating acceptable internal consistency. Overall, these findings suggest that the measurement instruments used in this study are reliable. The results are given in Table 2.

**TABLE 2:** Internal consistency coefficients of the scales.

Scale	Number of items	Cronbach's *α*
COPE	60	0.94
Childhood Trauma (CCTO)	53	0.96
SCL‐90‐R	90	0.98
ACY	5	0.78
Beck Depression Inventory	21	0.90
Rosenberg Self‐Esteem	12	0.82
SF‐36	36	0.88

Pearson correlation analysis showed that depression was strongly positively correlated with psychological symptoms (*r* = 0.76, *p* < 0.01). Childhood trauma was positively correlated with both depression (*r* = 0.32, *p* < 0.01) and psychological symptoms (*r* = 0.41, *p* < 0.01). Parental interest was also positively associated with childhood trauma (*r* = 0.33, *p* < 0.01) and depression (*r* = 0.37, *p* < 0.01). In addition, depression was negatively correlated with general health (SF‐36) (*r* = −0.22, *p* < 0.05). BMI was not significantly correlated with any of the psychological variables. The results are presented in Table 3.

**TABLE 3:** Correlations among study variables.

Variable	1	2	3	4	5	6	7
BMI	—						
COPE Total	0.05	—					
Childhood Trauma	0.11	0.15	—				
SCL‐90‐R	−0.00	0.23^*^	0.41^**^	—			
Depression	0.10	0.17	0.32^**^	0.76^**^	—		
SF‐36	−0.13	−0.12	−0.03	−0.09	−0.22^*^	—	
Parental Interest	0.15	0.15	0.33^**^	0.22	0.37^**^	−0.10	—

* *p* < 0.05.

** *p* < 0.01.

**FIGURE 1:**
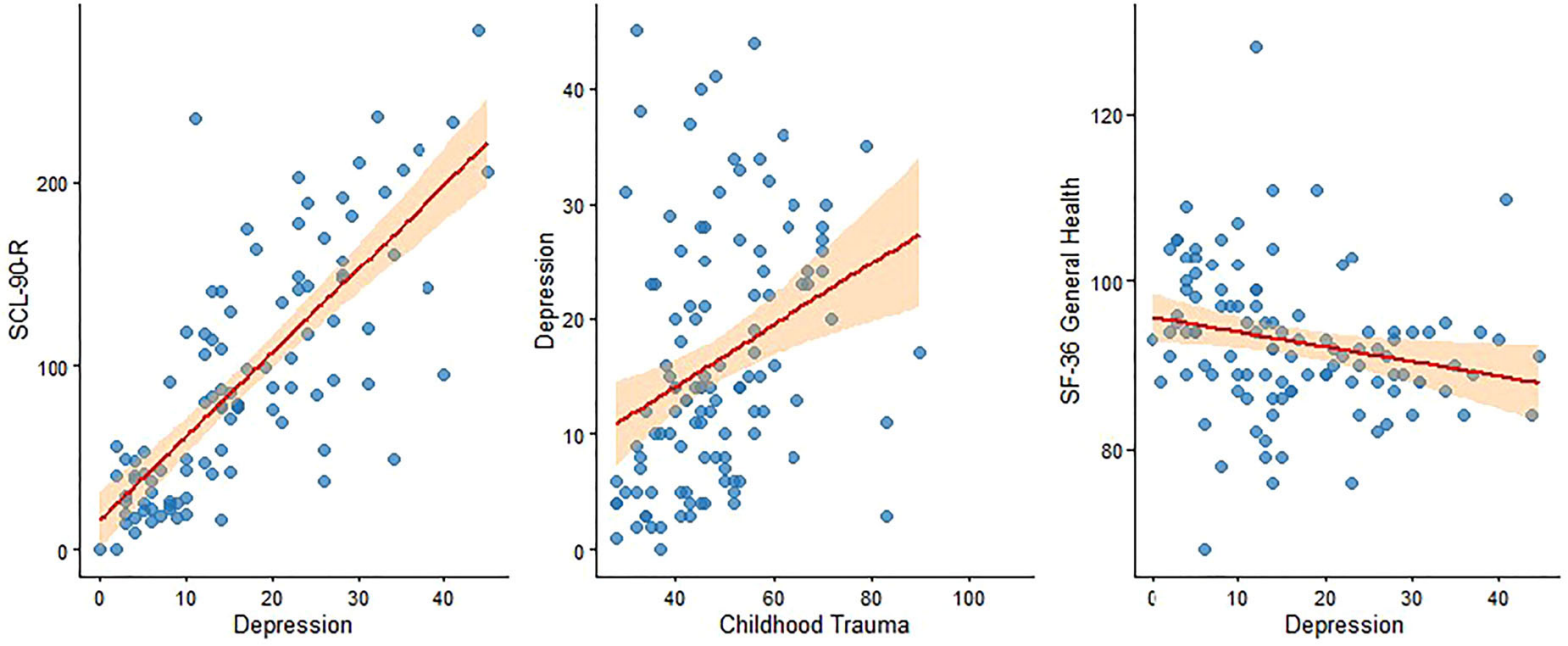
Scatter plots showing the relationships between depression and psychological symptoms (SCL‐90‐R), childhood trauma and depression, and depression and general health (SF‐36). The plots indicate a strong positive relationship between depression and psychological symptoms, a moderate positive relationship between childhood trauma and depression, and a negative relationship between depression and general health.

As shown in Figure 1, depression was strongly positively associated with psychological symptoms. In addition, childhood trauma was positively associated with depression. Furthermore, depression was negatively associated with general health, indicating that higher depression levels were related to poorer perceived health.

Multiple regression analysis showed that depression (*β* = 0.68, *p* < 0.001), childhood trauma (*β* = 0.29, *p* < 0.01), coping (*β* = 0.15, *p* < 0.05), and parental interest (*β* = −0.17, *p* < 0.05) significantly predicted psychological symptoms (SCL), and the model explained 66% of the variance. In addition, parental interest (*β* = 0.32, *p* < 0.01) was a significant predictor of depression, while childhood trauma and general health showed marginal effects (Table 4). Multicollinearity analysis indicated no multicollinearity problem among the independent variables.

**TABLE 4:** Multiple regression analyses predicting psychological symptoms and depression.

SCL (Model 1)							Depression (Model 2)					
Predictor	*B*	SE	*β*	*t*	*p*	VIF	*B*	SE	*β*	*t*	*p*	VIF
Intercept	−90.67	65.18		−1.39	0.169	—	16.03	14.87		1.08	0.284	—
Childhood Trauma	1.41	0.38	0.29	3.67	0.001^**^	1.28	0.14	0.09	0.17	1.67	0.098	1.15
Depression	4.33	0.48	0.68	9.07	< 0.001^**^	1.16	—	—	—	—	—	—
COPE	0.53	0.23	0.15	2.26	0.027^*^	1.06	0.04	0.05	0.08	0.79	0.429	1.03
Parental Interest	−1.81	0.85	−0.17	−2.14	0.036^*^	1.33	0.45	0.15	0.32	3.03	0.003^**^	1.17
SF‐36	−0.06	0.53	−0.01	−0.11	0.916	1.05	−0.22	0.12	−0.20	−1.77	0.081	1.02

*Note*: Unstandardized coefficients (*B*) are reported. Model 1: *R* ^2^ = 0.66, adjusted *R* ^2^ = 0.64, *F* = 26.90, *p* < 0.001. Model 2: *R* ^2^ = 0.24, adjusted *R* ^2^ = 0.20, *F* = 6.31, *p* < 0.001.

* *p* < 0.05 statistically significant.

** *p* < 0.01 statistically significant.

## 3 Discussion

This study aimed to cross‐sectionally characterize the psychological profiles of bariatric surgery candidates by examining the implications of childhood trauma, parental care, coping mechanisms, and depression. The findings reveal that bariatric surgery candidates are linked not only by issues related to weight gain but also by their past life histories and emotional processes. One of the most critical outcomes of our study is that BMI did not exhibit a statistically significant correlation with any of the psychological variables examined. Furthermore, this finding contrasts with the general expectations documented in the literature.

In our sample of bariatric surgery candidates, coping strategies were found to significantly predict general psychological symptoms. This indicates that the mechanisms deployed by our sample during stressful situations are associated with their overall psychological status. In contrast to this finding, coping mechanism scores did not exhibit a direct correlation with BMI. Based on this outcome, we suggest that there is no linear relationship between the severity of obesity and coping mechanisms among bariatric surgery candidates in our study. It can be argued that possessing a higher BMI does not inherently correlate with greater psychological distress. Furthermore, it can be stated that the psychological status of our sample is closely related to internal psychosocial factors and early life experiences rather than the weight problems they experience. In a 2019 study by Varela et al., a structural equation model demonstrated an indirect relationship between certain psychological states, such as coping attitudes, and BMI (Varela et al. 2020). Another study reported that coping attitudes exhibit an association with exerting an effect on BMI through their subsequent impact on eating behaviors (Aynehchi et al. 2023). However, an extremely important methodological caveat should be taken into account when interpreting this finding. The entire sample in our study consisted of bariatric surgery candidates with severe obesity. Therefore, the range of BMI was partially restricted. This situation may substantially attenuate the correlation coefficients. Another point that should be taken into consideration is that this restriction may reduce the statistical power required to detect significant relationships that could emerge in other sample groups ranging from normal weight to the most extreme classes of obesity. Studies in the literature have reported that there may not be a linear relationship between severe obesity and psychological burden and that psychological distress tends to plateau at extremely high BMI levels (McLaughlin and Hinyard 2014). This finding obtained from our study reveals that the diversity in psychological distress levels among the participants in our study is shaped by internal psychosocial dynamics.

It can be stated that the multiple linear regression model conducted in our study explained a substantially high proportion of the variance in general psychological symptoms. Depression was the independent variable that most strongly predicted general psychological symptoms. Together with this finding, it can be argued that within our study sample, depression is not merely a comorbid symptom but rather a central clinical hub embedded within the overarching psychopathological process. Furthermore, it was observed within the model that as depression levels increased, general health perception decreased. This situation suggests that individuals evaluate their physical status through an emotional lens. However, when interpreting the significant predictive power of depressive symptoms on general psychopathology, an important psychometric caveat should be taken into consideration. The significant relationship between the scores obtained from the SCL‐90‐R and BDI scales may partly reflect shared conceptual variance and measurement overlap. Through this shared variance, it can be suggested that depressive affect lies at the core of the psychological distress framework of the bariatric surgery candidates included in our study. However, in making this interpretation, it should be noted that natural psychometric overlap may occur, and it is recommended that this statistical relationship not be interpreted as reflecting entirely distinct clinical phenomena.

One of the most striking findings of the present study is that 83.8% of the bariatric surgery candidates reported a history of at least one suicide attempt. This variable was assessed retrospectively using a single item embedded within the Sociodemographic and Clinical Information Form, which was developed by the researchers and administered to the participants. Within this form, participants were asked the question: “Have you ever attempted suicide at least once in your life?” Affirmative responses were classified as indicating a history of at least one suicide attempt. No subjective details, such as method, intent, or degree of medical lethality, were assessed in this context. A review of the literature indicates that such prevalence rates vary across studies. For instance, in a 2022 study conducted by Sağaltıcı et al., the lifetime prevalence of suicidal ideation was reported as 23.7%, whereas the rate of previous suicide attempts was 13.5% (Sağaltıcı et al. 2022). In another study, 25% of bariatric surgery patients reported a lifetime history of preoperative suicidal ideation, while 4% reported a history of suicide attempts (Gordon et al. 2019). Furthermore, a study by Chen et al. focused on bariatric surgery candidates and demonstrated that 30.3% of the participants had a history of suicidal ideation (Chen et al. 2012). Taken together, the findings obtained in our study are significantly higher than the baseline prevalence rates reported in the literature for populations with obesity and patients undergoing bariatric surgery. It can be argued that preexisting psychiatric comorbidity before bariatric surgery—particularly impulsivity—constitutes a major underlying vulnerability factor for postoperative suicide risk (Mitchell et al. 2013). Moreover, large‐scale cohort studies have documented that patients with obesity who undergo bariatric surgery exhibit higher rates of suicidal ideation, non‐suicidal self‐injury, and completed suicide than the general population (Kauppila et al. 2020; Peterhänsel et al. 2013). In light of these data, it can be concluded that the patients with severe obesity in our sample who sought surgical intervention experienced profound psychological vulnerability in the preoperative period. On the other hand, methodological background must be taken into rigorous consideration when evaluating this specific finding. The data in question were not captured using a standardized clinical psychometric scale; instead, they were screened via a single self‐report question within a sociodemographic questionnaire. Consequently, participants might have responded to this item based on subjective interpretations, potentially subsuming behaviors such as non‐suicidal self‐injury lacking definitive lethal intent or intense suicidal ideation under the umbrella of a “suicide attempt.” Therefore, this subjective interpretation may have acted as an operational factor contributing to a prevalence rate substantially higher than those typically reported in the literature.

The findings obtained from this study reveal that childhood trauma experiences among bariatric surgery candidates are significantly associated with depression and general psychological symptoms. In light of these outcomes, it can be argued that bariatric surgery candidates must be addressed not only through physical dimensions but also through psychosocial ones. Prior research has demonstrated that adversities originating from the family environment and childhood traumas lead to a deficiency in protective mechanisms, thereby rendering individuals more vulnerable to psychological distress (Siegenthaler et al. 2012). Consequently, investigators have stated that encountering issues such as eating disorders and obesity becomes inevitable (Hemmingsson 2014). These findings from our study, alongside previous literature, indicate that adverse experiences, particularly during childhood, are associated with changes in neuroendocrine response systems and thus relate to impairments in problem‐solving skills. In addition, in the regression model constructed in our study, it was determined that both childhood trauma scores and depressive symptom levels statistically significantly and concurrently predicted overall psychological symptom severity. Cumulative research from the past to the present establishes that traumas experienced during childhood are linked to the emotional and cognitive development of individuals. Moreover, these experiences emerge as a robust predictor of psychological symptoms in adulthood. Studies conducted with individuals with clinical obesity have yielded results that support the findings of our study (Paone et al. 2024).

The psychological vulnerability identified among the bariatric surgery candidates in our study is of considerable importance not only in the preoperative period but also in terms of postoperative quality of life and psychosocial adjustment. Studies in the literature have shown that processes related to body contouring both renew body image and create positive effects on general psychosocial well‐being (Toto et al. 2025). Therefore, the surgical process, weight loss, other postoperative physical needs, and preoperative severe psychological distress should be addressed in a holistic manner. To this end, taking all of these factors into account in bariatric surgery candidates holds an important place in improving individuals' quality of life in the long term.

In the model evaluating the predictors of depression variance in our study, parenting attitudes emerged as the only statistically significant predictor. Studies investigating the relationship between parenting attitudes and obesity have indicated significant associations between parental indifference and the risk of developing obesity in adulthood (Bahrami et al. 2013; Amianto et al. 2016; Anderson et al. 2012). Early unresponsive and indifferent parenting attitudes share a similar mechanism with childhood traumas regarding obesity risk (Akers et al. 2025). It is known that such attitudes manifest as emotional and behavioral dysregulation during adulthood and present as negative schemas, such as disorganized attachment (Akers et al. 2025). Consequently, parental indifference toward children is associated with the emergence of early negative affectivity, and in later periods, psychological problems are closely associated with obesity (Roettger et al. 2023). On the other hand, it has been reported that depression and various psychological problems generally correlate negatively with psychological flexibility, showing an association with a high prevalence of unhealthy dietary habits and sedentary lifestyle conditions (Burhan et al. 2024). This phenomenon can be discussed through stress hormone mechanisms; specifically, the regulation of appetite and metabolism is linked to an increase in cortisol levels (Faith et al. 2011). Furthermore, impairments in quality of life due to various factors are linked to situations such as decreased physical activity and social isolation, which co‐occur with an increased risk of obesity (Weinberger et al. 2018). In our study, parenting attitude and parental care emerged as two of the strongest predictors of depression. Conversely, a 2018 study by Park and Kim found that negative parental experiences during childhood did not robustly predict depressive symptoms in adulthood (Park and Kim 2018). However, a study conducted with a general population sample demonstrated that parenting attitudes predict depression (Ishii et al. 2021). Similar to the aforementioned studies, our results support the view that insufficient parental care is linked to emotional dysregulation and obesity‐related psychological problems in adulthood.

Another remarkable finding among the results of our study is that parental care and parenting attitudes, which were expected to predict general psychological symptoms, inverted their direction and received a negative coefficient. With the inclusion of childhood trauma and depression, which can be designated as major variables, into the model, the association of parenting attitudes becomes complex. Therefore, it is possible to suggest that it exerts an indirect rather than a direct predictive role on general psychological symptoms. There are studies reporting that the direct association disappears when variables accounting for the impact of parental care on depression are controlled (Amianto et al. 2016). These disparate results observed across the literature and our study may stem from sample characteristics, cultural influences, measurement modalities, and the diversity of variables included in the model. Another plausible explanation points toward the cross‐sectional design of our study. Due to the cross‐sectional nature of our research, these findings should not be interpreted within a causal framework. These results should be evaluated as a robust pattern of concurrent common variance among the variables. It is highly probable that studies conducted with larger sample sizes will yield different outcomes regarding these variables.

In addition to the robust and significant findings of our study, several limitations must be acknowledged. First, the study utilizes a cross‐sectional design, which complicates the interpretation of the findings in terms of causality. Given that the variables investigated are subject to change over time, it is highly valuable for future research to support these models with longitudinal designs. Furthermore, the assessment instruments employed in this study are self‐report scales, which may present a limitation regarding the generalizability of the results. Another limitation is the lack of comparative groups with distinct characteristics; therefore, future studies are recommended to investigate similar models using healthy samples or populations with varying BMIs. Although our study reached a borderline sample size relative to the statistical analyses performed, employing larger sample cohorts in prospective research could enhance model stability. In addition, the participants were recruited from a single center, and cultural, socioeconomic, and environmental factors were not controlled. The sample is also predominantly female, which weakens its power to fully represent the broader population. In future studies, evaluating the effects of these variables across different dynamics would be of significant value. Finally, a major remaining limitation of our study is the exclusion of certain confounding factors that could directly influence both BMI and psychological health. The analyses performed in our study were exploratory in nature, and the findings are not confirmatory. Therefore, our results should be interpreted as hypothesis‐generating. Furthermore, it is recommended that these findings be replicated in future studies utilizing distinct hypotheses.

It is important to note that key variables central to our study, such as childhood trauma and parental attitudes, were assessed using retrospective self‐report scales. In this methodological approach involving the use of such self‐report scales, the potential influence of recall bias and memory processes may come to the forefront. Some studies have indicated that current psychological and emotional states can influence the evaluation of childhood experiences during adulthood (Hardt and Rutter 2004). Accordingly, participants in our study with higher levels of psychological distress may have had more negative retrospective perspectives and may have evaluated their childhood experiences and parental care accordingly. Therefore, to eliminate such limitations and obtain more accurate results, it is recommended that future studies adopt a longitudinal design.

In light of the particularly high prevalence rates observed in the present study, most notably with regard to the history of suicide attempts, the use of standardized psychometric instruments is strongly recommended for future prospective investigations. Such an approach would enable subsequent studies to address not only the presence of a history of suicide attempts, but also more specific dimensions, including non‐suicidal self‐injury, suicidal ideation, intent, method, and medical lethality, thereby allowing for a more comprehensive and detailed elucidation of the significance of suicide risk within this population.

## 4 Conclusion

In conclusion, this study reflects the multidimensional nature of psychological processes in bariatric surgery candidates and the potential reciprocal associations among these factors. Childhood traumas, low quality of life, symptoms of psychological distress, and early parental indifference emerge as prominent components of the psychological framework in bariatric surgery candidates. The prevalence of a history of suicide attempts was notably high among the sample included in the present study. Accordingly, it may be argued that psychiatric evaluation before surgical intervention, along with a systematic assessment of suicide risk, represents a critical component of preoperative care for bariatric surgery candidates. Coupled with this finding, the high rates of unemployment and low socioeconomic status underscore the critical importance of referring these patients during the preoperative period not only for nutritional counseling but also for comprehensive, multidisciplinary psychological screening and intervention programs. Consequently, these factors must be systematically integrated into both the prevention and the multidimensional management of obesity. Orchestrating this process through a combination of biological interventions, trauma‐informed therapeutic approaches, psychosocial support networks, and family‐based practices will offer an essential paradigm for achieving optimal clinical outcomes.

## Author Contributions

**Dilek Baysal**: conceptualization, formal analysis, resources, methodology, Writing – original draft. **Hakkı Polat**: conceptualization, formal analysis, methodology, writing – original draft. **Şafak Yalçin Şahiner**: conceptualization, investigation, methodology, project administration, supervision, writing – original draft, writing – review and editing. **Abdurrahim Bakirhan**: data curation, supervision, writing – review and editing.

## Funding

The authors have nothing to report.

## Ethics Statement

The study was conducted in accordance with the principles of the Helsinki Declaration and was approved by the Clinical Research Ethics Committee of Ankara Numune Training and Research Hospital (Approval number: E‐19‐2665, Date: April 18, 2019)

## Consent

Informed consent was obtained from all participants.

## Conflicts of Interest

The authors declare no conflicts of interest.

## Data Availability

The datasets used in this study were obtained from patients who provided informed consent, with their names and personal information kept strictly confidential. These datasets contain de‐identified information. Due to ethical and legal restrictions, as well as the potential risk of re‐identification of participants, the data cannot be publicly shared. However, upon reasonable request and subject to the approval of the corresponding author, de‐identified analytical data may be made available, provided that appropriate institutional and ethical permissions are obtained.

## References

[brb371750-bib-0001] Akers, N. , C. D. J. Taylor , and K. Berry . 2025. “Understanding the Relationships Between Parenting, Attachment, Schemas and Psychosis: A Serial Mediation Analysis.” British Journal of Clinical Psychology 64, no. 4: 939–963. 10.1111/bjc.12545. 40346947 PMC12506944

[brb371750-bib-0002] Amianto, F. , R. Ercole , G. Abbate Daga , and S. Fassino . 2016. “Exploring Parental Bonding in BED and Non‐BED Obesity Compared With Healthy Controls: Clinical, Personality and Psychopathology Correlates.” European Eating Disorders Review 24, no. 3: 187–196. 10.1002/erv.2419. 26603379

[brb371750-bib-0003] Anderson, S. E. , R. A. Gooze , S. Lemeshow , and R. C. Whitaker . 2012. “Quality of Early Maternal–Child Relationship and Risk of Adolescent Obesity.” Pediatrics 129, no. 1: 132–140. 10.1542/peds.2011-0972. 22201144 PMC3255468

[brb371750-bib-0004] Aynehchi, A. , S. Saleh‐Ghadimi , and P. Dehghan . 2023. “The Association of Self‐Efficacy and Coping Strategies With Body Mass Index Is Mediated by Eating Behaviors and Dietary Intake Among Young Females: A Structural‐Equation Modeling Approach.” PLoS ONE 18, no. 1: e0279364. 10.1371/journal.pone.0279364. 36706081 PMC9882783

[brb371750-bib-0005] Bahrami, F. , R. Kelishadi , N. Jafari , Z. Kaveh , and O. Isanejad . 2013. “Association of Children's Obesity With the Quality of Parental–Child Attachment and Psychological Variables.” Acta Paediatrica 102, no. 7: e321–e324. 10.1111/apa.12253. 23600901

[brb371750-bib-0006] Beck, A. T. , C. H. Ward , M. Mendelson , J. Mock , and J. Erbaugh . 1961. “An Inventory for Measuring Depression.” Archives of General Psychiatry 4: 561–571. 10.1001/archpsyc.1961.01710120031004. 13688369

[brb371750-bib-0007] Ben‐Porat, T. , S. Bacon , R. Woods , A. Fortin , and K. Lavoie . 2023. “Childhood Maltreatment in Patients Undergoing Bariatric Surgery: Implications for Weight Loss, Depression and Eating Behavior.” Nutrients 15, no. 9: 2046. 10.3390/nu15092046. 37432188 PMC10181145

[brb371750-bib-0008] Bernstein, D. P. , L. Fink , L. Handelsman , et al. 1994. “Initial Reliability and Validity of a New Retrospective Measure of Child Abuse and Neglect.” American Journal of Psychiatry 151, no. 8: 1132–1136. 10.1176/ajp.151.8.1132. 8037246

[brb371750-bib-0009] Bernstein, D. P. , J. A. Stein , M. D. Newcomb , et al. 2003. “Development and Validation of a Brief Screening Version of the Childhood Trauma Questionnaire.” Child Abuse & Neglect 27, no. 2: 169–190. 10.1016/s0145-2134(02)00541-0. 12615092

[brb371750-bib-0010] Burhan, Ş. , S. İ. Azizoğlu , T. Kuru , and H. Ş. Burhan . 2024. “Psychological Inflexibility and Obesity: Mediating Factors in Psychological Health.” Experimental Biomedical Research 7, no. 2: 94–102. 10.30714/j-ebr.2024.212.

[brb371750-bib-0011] Carver, C. S. , M. F. Scheier , and J. K. Weintraub . 1989. “Assessing Coping Strategies: A Theoretically Based Approach.” Journal of Personality and Social Psychology 56, no. 2: 267–283. 10.1037//0022-3514.56.2.267. 2926629

[brb371750-bib-0012] Chen, E. Y. , K. C. Fettich , and M. S. McCloskey . 2012. “Correlates of Suicidal Ideation and/or Behavior in Bariatric‐Surgery‐Seeking Individuals With Severe Obesity.” Crisis 33, no. 3: 137–143. 10.1027/0227-5910/a000115. 22343060

[brb371750-bib-0013] Chung, K. , H. Chiou , and Y. Chen . 2015. “Psychological and Physiological Correlates of Childhood Obesity in Taiwan.” Scientific Reports 5, no. 1: 17439. 10.1038/srep17439. 26612264 PMC4661724

[brb371750-bib-0014] Çuhadaroğlu, F. 1986. Self‐Esteem in Adolescents. Hacettepe University Faculty of Medicine.

[brb371750-bib-0015] Dağ, İ. 1991. “Reliability and Validity of the Symptom Check List (SCL‐90‐R) for University Students.” Turkish Journal of Psychiatry 2: 5–12.

[brb371750-bib-0016] Danese, A. , and M. Tan . 2014. “Childhood Maltreatment and Obesity: Systematic Review and Meta‐Analysis.” Molecular Psychiatry 19, no. 5: 544–554. 10.1038/mp.2013.54. 23689533

[brb371750-bib-0017] Derogatis, L. R. , K. Rickels , and A. F. Rock . 1976. “The SCL‐90 and the MMPI: A Step in the Validation of a New Self‐Report Scale.” British Journal of Psychiatry 128: 280–289. 10.1192/bjp.128.3.280. 1252693

[brb371750-bib-0018] Dicle, A. D. , and K. Ersanlı . 2015. “Turkish Standardization and the Validity and Reliability Studies of the Cope.” Journal of Academic Social Sciences 3, no. 16: 111–126.

[brb371750-bib-0019] Di Nicola, M. , M. R. Magurano , M. Pepe , et al. 2025. “The Association Between Childhood Trauma, Emotional Dysregulation, and Depressive Symptoms' Severity in Patients With Obesity Seeking Bariatric Surgery.” Journal of Personalized Medicine 15, no. 7: 303. 10.3390/jpm15070303. 40710420 PMC12299460

[brb371750-bib-0020] Faith, M. S. , M. Butryn , T. A. Wadden , A. Fabricatore , A. M. Nguyen , and S. B. Heymsfield . 2011. “Evidence for Prospective Associations Among Depression and Obesity in Population‐Based Studies.” Obesity Reviews 12, no. 5: e438–e453. 10.1111/j.1467-789X.2010.00843.x. 21414128

[brb371750-bib-0021] Giel, K. E. , S. Zipfel , R. Schweizer , et al. 2013. “Eating Disorder Pathology in Adolescents Participating in a Lifestyle Intervention for Obesity: Associations With Weight Change, General Psychopathology and Health‐Related Quality of Life.” Obesity Facts 6, no. 4: 307–316. 10.1159/000354534. 23941969 PMC5644671

[brb371750-bib-0022] Gordon, K. H. , W. C. King , G. E. White , et al. 2019. “A Longitudinal Examination of Suicide‐Related Thoughts and Behaviors Among Bariatric Surgery Patients.” Surgery for Obesity and Related Diseases 15, no. 2: 269–278. 10.1016/j.soard.2018.12.001. 31010651 PMC6481310

[brb371750-bib-0023] Hardt, J. , and M. Rutter . 2004. “Validity of Adult Retrospective Reports of Adverse Childhood Experiences: Review of the Evidence.” Journal of Child Psychology and Psychiatry 45, no. 2: 260–273. 10.1111/j.1469-7610.2004.00218.x. 14982240

[brb371750-bib-0024] Hemmingsson, E. 2014. “A New Model of the Role of Psychological and Emotional Distress in Promoting Obesity: Conceptual Review With Implications for Treatment and Prevention.” Obesity Reviews 15, no. 9: 769–779. 10.1111/obr.12197. 24931366

[brb371750-bib-0025] Hisli, N. 1988. “A Study on the Validity of the Beck Depression Inventory.” Turkish Psychological Journal 6, no. 22: 118–126.

[brb371750-bib-0026] Ishii, Y. , J. Masuya , C. Morishita , M. Higashiyama , T. Inoue , and M. Ichiki . 2021. “Victimization in Childhood Mediates the Association Between Parenting Quality, Stressful Life Events, and Depression in Adulthood.” Neuropsychiatric Disease and Treatment 17: 3171–3182. 10.2147/NDT.S323592. 34703237 PMC8541763

[brb371750-bib-0027] Kauppila, J. H. , G. Santoni , W. Tao , et al. 2020. “Risk Factors for Suicide After Bariatric Surgery in a Population‐Based Nationwide Study in Five Nordic Countries.” Annals of Surgery 275, no. 2: e410–e414. 10.1097/sla.0000000000004232. 32657942

[brb371750-bib-0028] Koçyiğit, H. , O. ¨. Aydemir , G. Fişek , N. Ölmez , and A. K. Memis . 1999. “Reliability and Validity of the Turkish Version of Short Form‐36 (SF‐36).” Turkish Journal of Drugs and Therapeutics 12: 102–106.

[brb371750-bib-0029] McLaughlin, L. , and L. J. Hinyard . 2014. “The Relationship Between Health‐Related Quality of Life and Body Mass Index.” Western Journal of Nursing Research 36, no. 8: 989–1001. 10.1177/0193945913520415. 24473057

[brb371750-bib-0030] Mitchell, J. E. , R. Crosby , M. de Zwaan , et al. 2013. “Possible Risk Factors for Increased Suicide Following Bariatric Surgery.” Obesity 21: 665–672. 10.1002/oby.20066. 23404774 PMC4372842

[brb371750-bib-0031] Mond, J. M. , B. Rodgers , P. J. Hay , et al. 2007. “Obesity and Impairment in Psychosocial Functioning in Women: The Mediating Role of Eating Disorder Features.” Obesity 15, no. 11: 2769–2779. 10.1038/oby.2007.329. 18070768

[brb371750-bib-0032] Offer, S. , E. Alexander , K. Barbara , E. Hemmingsson , S. W. Flint , and B. J. Lawrence . 2022. “The Association Between Childhood Trauma and Overweight and Obesity in Young Adults: The Mediating Role of Food Addiction.” Eating and Weight Disorders—Studies on Anorexia, Bulimia and Obesity 27, no. 8: 3257–3266. 10.1007/s40519-022-01454-y. PMC9803752 35907144

[brb371750-bib-0033] Orak, O. S. , H. İ. Bilkay , and Ç. Zengin . 2023. “Effect of Childhood Trauma on Substance Users' Attitudes of Coping With Stress.” Journal of Dependence 24, no. 3: 305–315. 10.51982/bagimli.1168435.

[brb371750-bib-0034] Paone, E. , M. Di Trani , E. Visani , et al. 2024. “Childhood Traumatic Experiences in People With Obesity With and Without Eating Disorders Who Are Seeking Bariatric Surgery: The Role of Attachment Relationships and family Functioning.” Eating and Weight Disorders—Studies on Anorexia, Bulimia and Obesity 29, no. 1: 9. 10.1007/s40519-024-01638-8. PMC10803430 38253926

[brb371750-bib-0035] Park, A. , and Y. Kim . 2018. “The Longitudinal Influence of Child Maltreatment on Child Obesity in South Korea: The Mediating Effects of Low Self‐Esteem and Depressive Symptoms.” Children and Youth Services Review 87: 34–40. 10.1016/j.childyouth.2018.02.012.

[brb371750-bib-0036] Peterhänsel, C. , D. Petroff , G. Klinitzke , A. Kersting , and B. Wagner . 2013. “Risk of Completed Suicide After Bariatric Surgery: A Systematic Review.” Obesity Reviews 14, no. 5: 369–382. 10.1111/obr.12014. 23297762

[brb371750-bib-0037] Quek, Y. H. , W. W. S. Tam , M. W. B. Zhang , and R. C. M. Ho . 2017. “Exploring the Association Between Childhood and Adolescent Obesity and Depression: A Meta‐Analysis.” Obesity Reviews 18, no. 7: 742–754. 10.1111/obr.12535. 28401646

[brb371750-bib-0038] Roettger, M. E. , B. Houle , and J. D. Boardman . 2023. “Parental Imprisonment, Delinquent Behavior, and BMI Gain in a U.S. Nationally Representative Cohort Study of Adolescents and Adults Ages 12–32.” SSM—Population Health 22: 101425. 10.1016/j.ssmph.2023.101425. 37215156 PMC10193003

[brb371750-bib-0039] Rosenberg, M. 1965. “Rosenberg Self‐Esteem Scale (RSE). Acceptance and Commitment Therapy.” Measures Package 61, no. 52: 18. 10.1037/t01038-000.

[brb371750-bib-0040] Sağaltıcı, E. , C. Ural , H. Belli , M. Akbudak , and Ş. Şahin . 2022. “Predictors of Binge Eating Disorder and the Impact on the Quality of Life in Patients With Severe Obesity Before Bariatric Surgery.” Journal of Contemporary Medicine 12, no. 2: 249–254. 10.16899/jcm.1011364.

[brb371750-bib-0041] Şar, V. , P. E. Öztürk , and E. İkikardeş . 2012. “Validity and Reliability of the Turkish Version of Childhood Trauma Questionnaire.” Turkiye Klinikleri Journal of Medical Sciences 32, no. 4: 1054–1063.

[brb371750-bib-0042] Siegenthaler, E. , T. Munder , and M. Egger . 2012. “Effect of Preventive Interventions in Mentally Ill Parents on the Mental Health of the Offspring: Systematic Review and Meta‐analysis.” Journal of the American Academy of Child and Adolescent Psychiatry 51, no. 1: 8–17.e8. 10.1016/j.jaac.2011.10.018. 22176935

[brb371750-bib-0043] Spirou, D. , J. Raman , R. H. Bishay , G. Ahlenstiel , and E. Smith . 2022. “Childhood Trauma, Posttraumatic Stress Disorder Symptoms, Early Maladaptive Schemas, and Schema Modes: A Comparison of Individuals With Obesity and Normal Weight Controls.” BMC Psychiatry 22, no. 1: 517. 10.1186/s12888-022-04169-7. 35907801 PMC9339192

[brb371750-bib-0044] Toto, V. , A. Faiola , M. Pazzaglia , et al. 2025. “Impact of Abdominoplasty With Diastasis Recti Correction on Female Sexual Function: A 1‐Year Prospective Follow‐Up Study.” Aesthetic Plastic Surgery 49, no. 17: 4925–4931. 10.1007/s00266-025-05002-8. 40634763

[brb371750-bib-0045] Varela, C. , A. Andrés , and C. Saldaña . 2020. “The Behavioral Pathway Model to Overweight and Obesity: Coping Strategies, Eating Behaviors and Body Mass Index.” Eating and Weight Disorders—Studies on Anorexia, Bulimia and Obesity 25, no. 5: 1277–1283. 10.1007/s40519-019-00760-2. 31376111

[brb371750-bib-0046] Ware, J. E. Jr. , and C. D. Sherbourne . 1992. “The MOS 36‐ltem Short‐Form Health Survey (SF‐36).” Medical Care 30, no. 6: 473–483. 10.1097/00005650-199206000-00002. 1593914

[brb371750-bib-0047] Weinberger, N. A. , A. Kersting , S. G. Riedel‐Heller , and C. Luck‐Sikorski . 2018. “The Relationship Between Weight Status and Depressive Symptoms in a Population Sample With Obesity: The Mediating Role of Appearance Evaluation.” Obesity Facts 11, no. 6: 514–523. 10.1159/000492000. 30554212 PMC6341343

[brb371750-bib-0048] World Obesity Federation . 2025. World Obesity Atlas 2025 . https://data.worldobesity.org/publications/?cat=23.

[brb371750-bib-0049] Zuckerman, M. , and M. Gagne . 2003. “The COPE Revised: Proposing a 5‐factor Model of Coping Strategies.” Journal of Research in Personality 37: 169–204. 10.1016/S0092-6566(02)00563-9.

